# Thyroid hormone concentrations associated with age, sex, reproductive status and apparent reproductive failure in the Amazon river dolphin (*Inia geoffrensis*)

**DOI:** 10.1093/conphys/coz041

**Published:** 2019-08-01

**Authors:** T R Robeck, R S Amaral, V M F da Silva, A R Martin, G A Montano, J L Brown

**Affiliations:** 1 Species Preservation Laboratory, SeaWorld Parks and Entertainment, 2595 Ingraham Rd, San Diego, CA 92109, USA; 2 Federal Institute of Education, Science and Technology of Amazonas—IFAM/CMZL, Av. Cosme Ferreira 8045, Manaus 69086-475, Brazil; 3 Laboratory of Aquatic Mammals, National Institute of Amazonian Research—INPA, Av Andre Araujo 2936, Manaus 69067-375, Brazil; 4 Centre for Remote Environments, University of Dundee, Nethergate, Dundee DD1 4HN, UK; 5 Center for Species Survival, Smithsonian Conservation Biology Institute, 1500 Remount Road, Front Royal, VA 22630, USA

**Keywords:** Boto, fetal demise, neonatal loss, river dolphins, thyroxine, triiodothyronine

## Abstract

This study was conducted to characterize immunoreactive thyroid hormone concentrations in wild Amazon river dolphins, also called boto (*Inia geoffrensis*) by age group, sex, pregnancy and lactation status, and to determine if thyroid hormone concentration differences could be detected between pregnant females with and without successful parturition outcomes. Radioimmunoassays were used to analyse total *T*_3_ and total *T*_4_ in 182 serum samples collected from 172 botos living in the Mamirauá Sustainable Development Reserve, in the Brazilian Amazon from 2003 through 2015. Age significantly affected t*T*_3_ and t*T*_4_ concentrations in males, with values in immature males and females being significantly lower than those in adult males, whereas no age effects were noted between immature females and adult non-pregnant, non-lactating females. Significant sex differences were noted in t*T*_3_ concentrations between immature males and females and in t*T*_4_ concentrations between adult males and females. These resulted in significant differences in the t*T*_3_:t*T*_4_ ratio between males and females within the immature and adult groups. Lactating and non-pregnant adult females had significantly higher t*T*_3_ concentrations than pregnant females, and this difference was primarily driven by a 12% drop in t*T*_3_ concentrations during the last two-thirds of pregnancy. No differences in thyroid hormone concentrations were detected between females diagnosed as pregnant and later found to have or not have a live calf. These results are the first to define thyroid hormone reference intervals and normal physiological variations in a wild population of river dolphins.

## Teaser

Thyroid hormones play important roles in metabolic and reproductive function; however, they have not been described in detail for any river dolphin species. We characterize these hormones across different age, sex and pregnancy status in the Amazon river dolphin in order to provide baseline normal values for this increasingly threatened species.

## Introduction

Global freshwater habitats are often adjacent to or within dense and expanding human populations and are increasingly being degraded due to anthropogenic activity. As a result, freshwater megafauna species, including all species of river dolphins, are under direct pressures, and if they continue unchecked, it may result in their extinction (He *et al*., 2017; Huang *et al*., 2012; Williams *et al*., 2016). Currently, of an estimated 10 species of fresh water dolphins, six are listed as endangered, critically endangered or functionally extinct, two are vulnerable and two populations are considered “status unknown” (Braulik and Smith, 2017; He *et al*., 2017; Reeves *et al*., 2011). While it is clear that freshwater dolphins are at extreme risk, what is less understood is how anthropogenic pressures are affecting their survival. In addition, as apex predators, these species often directly compete with man for food, and many of the environmental factors that may be detrimental towards their overall health and well-being could also affect human populations. Thus, these species, and cetaceans in general, may represent one of the best environmental sentinels for human populations living nearby (Bossart, 2006; Chen *et al.,* 2017; Lailson-Brito *et al.,* 2008).

Despite the importance of river dolphins for habitat health (Turvey *et al.,* 2012), and as markers of adverse environmental threats, little is known about their physiology, or how various species may be affected by deteriorating conditions. The Amazon river dolphin, or boto (*Inia geoffrensis*), exists in habitats that are currently relatively free from industrial development and high population density (da Silva *et al.,* 2018). Despite this, recent evidence suggests this species is in decline due to anthropogenic pressures created from direct harvesting of the animals for use as bait to catch commercially valuable catfish, the piracatinga (*Calophysus macropterus*), by native fisherman (Mintzer *et al.,* 2013; da Silva *et al.,* 2018). In addition, multiple proposals exist for hydroelectric projects throughout the Amazon river basin that may threaten the population through habitat fragmentation (Finer and Jenkins, 2012). These current and future pressures may threaten boto sustainability, and therefore, there may be a limited time period during which physiologic health markers and physiological reference ranges can be defined and used as “normal” controls for future population health assessments.

Several health assessments of wild cetacean populations have been published over the last 20 years; the value of which has been demonstrated by recent research on wild bottlenose dolphin populations adversely impacted by a regional oil spill (Schwanke *et al.,* 2014). Data from both captive and wild healthy populations have enabled researchers to develop models identifying the adverse short- and long-term impacts from this oil spill (Hall *et al.,* 2018; Lane *et al.,* 2015; Smith *et al.,* 2017). Recently, health assessments of wild cetaceans have been applied to an increasing number of marine and freshwater cetacean species (Flower *et al.,* 2015; Nabi *et al.,* 2018; Smith *et al.,* 2017; Van Bressem *et al.,* 2009). Blood and other biological samples collected from animals during these assessments provided the opportunity to examine multiple organ and endocrine systems. For cetaceans, overfishing and climate change have elevated the importance of monitoring thyroid function via thyroid hormones to detect critical homeostatic changes in response to these external stressors (Fair *et al.,* 2011; Flower *et al.,* 2015; St. Aubin *et al.,* 1996; Wasser *et al.,* 2017). However, before detecting abnormal function, normal concentrations during varying life history events must be defined.

Thyroid hormones have been documented to vary by age and different physiological conditions, e.g. pregnancy and lactation, in multiple mammalian species, including cetaceans (Fair *et al.,* 2011; Flower *et al.,* 2015; St. Aubin, 2001; St. Aubin *et al.,* 1996; West *et al.,* 2014). Comparatively high hormone concentrations and proportionally large thyroid glands of marine cetaceans (Fair *et al.,* 2011; Ridgway and Patton, 1971) have led researchers to speculate that cold environmental temperatures combined with high heat conductivity of water habitats requires an increased metabolic response to maintain core body temperature, which is driven by enhanced thyroid activity. In addition, changes in thyroid hormone concentrations that parallel seasonal environment changes and prey availability have been described for belugas (*Delphinapterus leucas*) and killer whales (*Orcinus orca*; Flower *et al.,* 2015; Wasser *et al.,* 2017).

Multiple thyroid pathologies have been identified in marine cetaceans, including colloid depletion, fibrosis, thyroiditis and squamous cysts (Cowan and Tajima, 2006; Harrison, 1969; Schumacher *et al.,* 1993). Abnormal thyroid gland function during pregnancy either in response to iodine deficiency or due to primary thyroid pathology has been known to be associated with both fetal and placental growth abnormalities that can translate into a number of pathologies including abortion, premature birth, low birth weight and neonatal failure to thrive syndrome (Glinoer, 2007). While no reports of thyroid pathology exist for the boto, or other river dolphin species, the frequency of its occurrence in marine cetaceans warrants such an investigation.

In addition to direct effects of thyroid pathology, malnutrition has been indirectly linked to thyroid dysfunction, and if this deficiency occurs during pregnancy, fetal growth restriction and other developmental abnormalities may result. Some recent evidence in beef cows demonstrated that changes in the percentage of protein while maintaining normal energy content can affect fetal thyroid development and programming without any changes in circulating maternal thyroid hormone concentrations (Micke *et al.,* 2015). In humans, maternal malnutrition has resulted in normal *T*_4_, and significantly low *T*_3_ concentrations compared with controls (Mahajan, *et al.,* 2005). Recent attempts to use fecal thyroid hormone concentrations as an indicator of nutritional stress and subsequent fetal loss in the killer whale have provided some support for this premise (Wasser *et al.,* 2017). While a direct link between malnutrition, thyroid dysfunction and fetal or neonatal abnormalities remains elusive, evaluation of normal thyroid function in the boto would provide baseline data from which future effects of nutritional stress may be evaluated.

**Figure 1:**
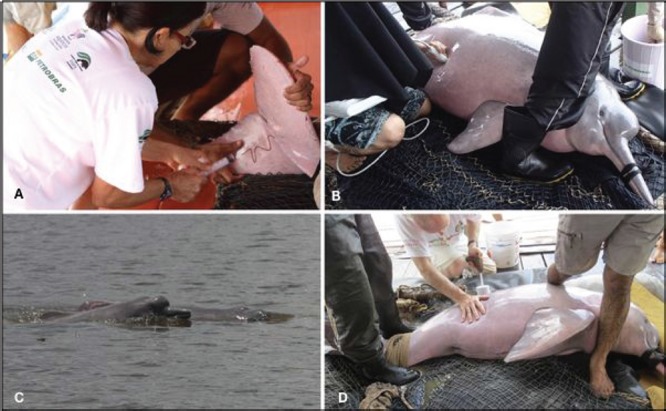
Health assessment on wild Amazon river dolphins (boto, *Inia geoffrensis*). Images depict blood sampling (a), ultrasound body examination (b), wild boto with calf (c) and freeze branding of animal prior to release (d). Images credited to Projeto Boto/INPA

Currently no significant *ex situ* populations of this species exist whereby thyroid hormone concentrations could be determined to establish baseline, and to our knowledge, only one report of thyroid hormone concentrations in wild botos exists. In that report, which analysed serum from eight wild animals, mean total thyroxine (t*T*_4_) concentration was determined without any information on sex, age or reproduction status of the animals that had been sampled (Ridgway *et al.,* 1970). Therefore, the goal of this research was to describe both t*T*_4_ and total triiodothyronine (t*T*_3_) concentrations in wild botos. Specific objectives were to (i) determine if concentrations of thyroid hormones are significantly affected by sex, age and female reproductive status; (ii) develop thyroid reference ranges for wild botos relative to sex, maturity and female reproductive status (non-pregnant nonlactating, pregnant and lactating); (iii) determine if thyroid hormone concentrations vary during trimesters of gestation; and (iv) determine if thyroid hormone concentrations in females diagnosed as pregnant differ between females who are subsequently observed to have had a live calf or are observed without a calf.

## Methods

### Animals and sample collections

A total of 182 samples was collected from 172 animals who were temporarily restrained during an annual capture–recapture campaign of Projeto Boto at the Mamirauá Sustainable Development Reserve, Brazilian Amazon, between 2003 and 2015. Once restrained, total length, sex, and reproductive status of adult females (non-pregnant, pregnant or lactating) was determined by a combination of visual inspection and transabdominal ultrasonography (Figure 1). Blood samples were collected from the ventral tail fluke using a 19-gauge winged blood collection set (Figure 1). Whole blood was collected into BD Vacutainers (Becton Dickenson, Franklin Lakes, NJ, USA) containing activated thrombin, allowed to clot for 20 min, and then centrifuged at 1000 g for 15 to 30 min. Serum was collected and stored at −20°C until analysis. Evidence indicates that minimal change in thyroid hormone concentrations occurs when stored at −25°C for up to 23 years (Männistö et al., 2007). For pregnancy determination, all examinations were performed using a Sonosite 180 Plus ultrasound unit (Pyramid Medical Systems, São Paulo, São Paulo, 04181-110, Brazil) in conjunction with a 2- to 5-MHz multi-frequency transducer (Convex Array 180 Plus/Elite-C60, Pyramid Medical Systems). Animals were determined to be pregnant by visualization of a conceptus (placental membranes, fetus) and uterine fluid (amniotic, chorionic or allantoic fluid). Fetal age was assigned to the first (0 to 112 days), second (113 to 224 days) or third trimester (225 to 333 days) of pregnancy using total length, biparital or transthoracic measurements, by reference to published measurements for bottlenose dolphins (*Tursiops truncatus*; Lacave *et al.,* 2004). Bottlenose dolphin fetal growth rates were chosen to estimate fetal age in boto due to the similarity in gestation length between the two species (Martin & da Silva, 2018). Based on serum testosterone in males (Amaral *et al.,* manuscript in prep), males were classified as immature (calf or juvenile males < 188 cm total body length) or adult (males ≥188 cm total body length). Females were classified based on shortest size at first pregnancy (Martin & da Silva, 2018); therefore, immature females were <180 cm total body length and adult females were ≥180 cm total body length. Adult females were further categorized based on physiological state—non-pregnant, pregnant, or lactating.

### Thyroid hormone radioimmunoassays

Thyroid hormones (total *T*_4_ and *T*_3_) were measured in serum using commercial solid phase radioimmunoassay (RIA) kits [Siemens Medical Solutions Diagnostics, Los Angeles, CA: total *T*_4_ (t*T*_4_) catalog number TKT45; total *T*_3_ (t*T*_3_) catalog number TKT35]. All RIAs were conducted in accordance with the manufacturer's instructions and were validated for use in dolphins based on observed parallelism between serial dilutions of pooled serum samples and the respective standard curves and >90% recovery of respective hormone standards from pooled serum samples. Assay sensitivities were 2.5 ng/ml for t*T*_4_, 0.1 pg/ml for f*T*_4_, 0.07 ng/ml for t*T*_3_ and 0.02 pg/ml for f*T*_3_. For all assays, intra- and inter-assay coefficients of variation were <15%. The relative percentage of total immunoreactive *T*_3_ and *T*_4_ were determined by dividing the mean value across all groups for each hormone, respectively, by the sum of both mean concentrations (μt*T*_3_ + μt*T*_4_ = total immunoreactive thyroid hormone concentrations).

### Statistical analysis

Data were analysed using Stata® (version 14; StataCorp LP, College Station, TX, USA). For females, to control for repeated samples from the same animal, and an unequal number of sampling among animals, we analysed data using a linear mixed effect restricted maximum likelihood regression model (West *et al.,* 2015) to quantify the relationship between the dependent variable (hormone concentration) and fixed effect variables, with animal ID as the random intercept variable with an unstructured covariance. The fixed effect categorical variables included age group (immature male and female, adult male and adult non-lactating, non-pregnant female), physiological state (pregnant, non-pregnant, non-pregnant lactating) and trimester (i.e. first trimester, second trimester, third trimester). Separate analyses were performed for each fixed effect variables and repeated for each dependent variable (i.e. hormone concentrations (t*T*_3_, t*T*_4_ and *T*_3_:*T*_4_ ratio).

All final mixed models were checked for normality using quantile plots of the standard residuals. If quantile–quantile (qnorm) plots of standardized residuals exhibited non-normal distribution, then data were transformed as indicated by the Shapiro–Wilk test (Ladder command, STATA) of raw data until model residuals were normalized. Pairwise comparisons of estimated marginal means were conducted using the margins command with Sidak correction or as paired contrasts without correction. Unless specified, data are expressed as back transformed marginal means and 95% confidence intervals. For all analyses, *P* < 0.05 was considered significant.

Comparisons of thyroid hormone concentration across gestational stages in pregnant females (as determined by ultrasonography during gestation) that had live calves versus pregnant females that did not have a live calf were determined by observations during the next annual field assessment. Since not all females were observed each year, only females which could be confirmed as having a calf within a 2-year period after they were diagnosed as pregnant (this length of time was used to accommodate for the various stages of gestation when they were initially assessed, and this the time interval to when a calf would have been born) were compared using unpaired two-tailed *t* test with unequal variances using the Welch (1947) approximation for degrees of freedom. Due to the length of time between examinations or observations of females we could not determine at which point the fetus or calf was lost. Therefore, the calf loss classification would include females that experienced an abortion, or stillbirth and calves that died during the perinatal period or that failed to thrive.

## Results

### Overall results

Across all sex and age groups, mean (±SD, 95% CI) t*T*_3_ and t*T*_4_ were 0.52 ± 0.15 ng/ml (0.49–0.55 ng/ml) and 38.6 ± 0.84 ng/ml (37.0–40.3 ng/ml), respectively. Based on mean concentrations, the relative percentage contribution towards total immunoreactive thyroid concentrations for t*T*_3_ and t*T*_4_ were 1.3% and 98.7%, respectively.

### Thyroid hormone reference ranges for the boto

Percentiles (2.5, 25, 50, 75, 97.5) for t*T*_3_, t*T*_4_ and the t*T*_3_:t*T*_4_ by age class and for adult female physiological status are shown in Table 1. Generally, lactating females had t*T*_3_ values that tended to be higher at all percentage cut-offs than the other female groups and adult males, while pregnant females consistently had the lowest concentrations. For t*T*_4_, juveniles of both sexes tended to have the greatest concentrations. Finally, and similarly for t*T*_3_, the t*T*_3_:t*T*_4_ ratio was greatest for lactating females and adult males.

**Table 1:** Serum thyroid hormone (total *T*_3_ ng/ml, total *T*_4_, *T*_3_:*T*_4_ ratio ×1000) reference concentrations for the Amazon river dolphin (*Inia geoffrensis*) in samples collected from 2003 to 2015

				Age class					
TH	Percentile	JF (n = 8)	JM (n = 23)	AM (n = 65)	AF (n = 26)	PF (n = 40)	1st (n = 15)	2nd (n = 21)	LF (n = 14)
t*T*_3_	2.5	0.28	0.38	0.25	0.13	0.24	0.27	0.24	0.31
	25	0.32	0.43	0.43	0.39	0.33	0.34	0.32	0.45
	50	0.46	0.57	0.49	0.50	0.39	0.44	0.37	0.57
	75	0.52	0.90	0.59	0.75	0.46	0.46	0.42	0.67
	97.5	0.71	1.53	1.05	1.1	0.76	0.76	0.53	0.94
t*T*_4_	2.5	31.7	23.1	20.7	16.5	15.8	15.6	20.8	14.7
	25	39.3	34.9	29.4	32.1	33.2	31.9	35.8	25.6
	50	44.1	42.0	33.1	39.7	38.2	42.7	37.7	36.0
	75	46.7	50.9	40.0	51.1	44.5	47	42.6	44.2
	97.5	51.2	68.7	51.5	96.7	61.5	61.8	50.3	67.8
*T*_3_:*T*_4_	2.5	6.0	7.5	7.2	4.7	5.4	5.4	6.5	4.6
	25	8.4	10.6	12.9	9.3	8.0	8.1	7.7	11.7
	50	10.2	13.6	15.2	11.9	10.2	11.1	10.0	15.6
	75	10.7	17.6	18.2	18.6	14.2	16.8	13.5	26.2
	97.5	12.3	32.9	27.7	23.0	24.7	24.7	14.4	44.2

JF: juvenille female, JM: juvenille male, AM: adult male, AF: adult female (nonpregnant, nonlactating), PF: pregnant female (pregnancy with resulting calf), 1st: first trimester, 2nd: combined second and third trimester, LF: lactating female.

**Table 2:** Comparisons of marginal mean (95% CI) total *T*_3_ (ng/ml), total *T*_4_ (ng/ml) and *T*_3_:*T*_4_ ratio (×1000) between sex within age groups (adult female vs adult male), between age groups within the same sex (juvenile vs adult) and in adult females during different physiologic states (pregnant, lactating, or non-lactating non-pregnant adult)

Group	t*T*_3_	t*T*_4_	t*T*_3_:t*T*_4_
Juvenile male (n = 23)	0.62 (0.54–0.71)	41.6 (37.7–46.0)	14.5 (12.4–16.9)
Juvenile female (n = 8)	0.43 (0.34–0.54)	42.6 (34.6–52.5)	9.4 (7.9–11.1)
Adult male (n = 65)	0.51 (0.47–0.55)	33.6 (31.7–35.5)	15.2 (14.2–16.2)
Adult female^a^ (n = 26)	0.49 (0.43–0.56)	38.9 (35.0–43.3)	12.2 (10.5–14.2)
Pregnant female^b^ (n = 40)	0.39 (0.35–0.43)	37.9 (34.8–41.4)	10.5 (9.4–11.8)
Lactating female^c^ (n = 14)	0.56 (0.47–0.67)	34.7 (28.6–42.1)	16.3 (12.0–16.9)
Pairwise comparisons of means^d^	IF < IM; AM < IM; PF < AF and LF	AM < IM AM < AF	IF < IM; AF < AM AF & PF < LF

^a^Non-pregnant, non-lactating adult females.

^b^Only females with “successful pregnancies” were included in this category. These were females which were diagnosed as pregnant and then were observed to have a calf the following season.

^c^Non-pregnant females.

^d^Only groups with differences (*P* < 0.05) were reported.

### Mean thyroid hormone concentrations between age and sex in boto

For age, the only significant differences in t*T*_3_ and t*T*_4_ concentrations were found between immature males (t*T*_3_: 0.62 ng/ml, t*T*_4_: 41.6 ng/ml) and adult males (t*T*_3_: 0.51 ng/ml, t*T*_4_: 33.6), whereas no differences in thyroid hormone concentrations between immature and adult non-pregnant, non-lactating females were detected (Table 2). Sex differences in t*T*_3_ were detected between immature males and immature females (0.66 ng/ml). For t*T*4, adult males (33.6 ng/ml) were significantly lower than adult non-lactating non-pregnant females (38.9 ng/ml; Table 2). And finally, the t*T*_3_:t*T*_4_ ratio was significantly higher for immature males compared with immature females and lower in adult females compared with adult males (Table 2).

### Mean thyroid hormone concentrations across female boto reproductive states

The concentrations of t*T*_3_ and t*T*_4_ and were similar during the second and third trimesters and were therefore combined for statistical analysis. Overall, t*T*_3_ was significantly lower in pregnant (0.39 ng/ml) compared with adult (non-pregnant, non-lactating) females. During pregnancy, females experienced a mean 12% decrease in t*T*_3_ from the first (0.42 ng/ml) to the combined second and third trimesters (0.37 ng/ml, Table 3). Therefore this decrease was primarily responsible for the overall significant reduction in t*T*_3_ during pregnancy (Table 3). Overall, pregnancy values were significantly lower than those in lactating females. For t*T*_4_, no significant differences were detected among the adult female groups, although lactating females had a significantly increased t*T*_3_:t*T*_4_ compared with all other adult female groups (Table 3).

**Table 3:** Comparisons of marginal mean (95% CI) total *T*_3_ (ng/ml), total *T*_4_ (ng/ml) and the *T*_3_:*T*_4_ ratio (×1000) during the first and combined second and third trimesters of pregnancy with non-pregnant, non-lactating, adult females and non-pregnant, lactating females

Group	t*T*_3_	t*T*_4_	t*T*_3_:t*T*_4_
Adult female (n = 26)	0.49 (0.43–0.56)	38.9 (35.0–43.3)	12.2 (10.5–14.2)	
First trimester (n = 17)	0.42 (0.35–0.49)	39.2 (34.1–44.9)	11.6 (9.5–14.0)	
Second and third trimester (n = 23)	0.37 (0.32–0.43)	37.3 (32.3–42.2)	9.7 (8.2–11.5)	
Lactating female (n = 14)	0.56 (0.47–0.67)	35.0 (29.5–41.7)	16.3 (13.1–20.4)	
Sidak Groups	Second and third < AF First, second, and third < LF	NSD	AF, first, second, and third < LF	

Females with confirmed calf loss either during gestation or after delivery were not included in this analysis.

NSD: no significant difference.

### Thyroid hormone concentrations in boto with successful versus non-successful pregnancies

Throughout gestation, no significant differences were detected in thyroid hormone concentrations between pregnant females that lost calves and those that successfully gave birth. In addition, no significant differences in thyroid hormone concentrations were found when comparing trimesters between normal and abnormal pregnancies.

## Discussion

### Boto thyroid hormone reference ranges

This study is the first report of thyroid hormone concentration reference ranges in relation to sex, age and reproductive status for any species of river dolphin and provides novel information regarding river dolphin physiology, species that are some of the least understood and most endangered of the cetaceans (He *et al.,* 2017). For example, it has been postulated that relatively high concentrations of *T*_4_ in marine cetaceans (beluga, bottlenose dolphins) compared with non-human terrestrial mammals may be an adaptation to the relatively cold climate associated with an aquatic environment (Fair *et al.,* 2011; Flower *et al.,* 2015). The reference range for t*T*_4_ in the boto supports this concept, as this cetacean species inhabits tropical waters that are considerably warmer than most encountered by other marine cetaceans (Sioli, 1984), and their concentrations were approximately half those of cold-water cetacean species. Further, t*T*_4_ concentrations appeared to be similar to terrestrial mammals (for review, see Fair *et al.,* 2011) and the semi tropical marine mammal manatee (*Trichechus manatus*, Ortiz et al., 2000).

In addition to adaptive variations, boto reference ranges provide evidence that thyroid hormone concentrations decrease with age in both males and females, similar to bottlenose dolphins (Fair *et al.,* 2011; West *et al.*, 2014) and belugas (Flower et al., 2015). A decrease in thyroid hormones with age has been observed in humans (Kapelari *et al.,* 2008; Lem *et al.,* 2012) and other mammals (Malinowski *et al.,* 1996) and may in part be explained by growth hormone, which stimulates peripheral *T*_3_ production in young animals (Lapierre *et al.,* 1990). Defining age specific changes in thyroid hormone concentrations allows for improved sensitivity for the detection of thyroid hormone deficiencies. When these deficiencies occur in juveniles, they can have profound effects on normal development; and therefore, defining the normal changes with age is an important component for the development of population health assessment criteria (Dwyer and Stickland, 1992; Lem *et al.,* 2012).

Normal changes or reference ranges within adult groups can be beneficial for identifying thyroid pathology—thyroiditis, hypo and hyper thyroidism—some of which can have profound effects on fetal and neonatal growth and development. While t*T*_4_ was relatively consistent among groups, t*T*_3_ and the t*T*_3_:t*T*_4_ ratio demonstrated large variation in concentration ranges, especially between pregnant and lactating females. In general, *T*_3_:*T*_4_ changes are used to reflect the efficiency of thyroid production and predict clinical disease, such as thyrotoxicosis, euthyroid sick syndrome, iodine deficiency, follicular cysts, thyroiditis and hyperthyroidism (Mortoglou and Candiloros, 2004). To our knowledge, most animals in this study were healthy at the time of sample collection, and combined with a fairly large sample size, these results should provide a robust reference interval that will be important for continued monitoring of individual animals and potentially provide a useful indicator for assessing potential changes in overall population health.

For the boto, the major thyroid hormone in serum is t*T*_4_ (98.7%) with only ~ 1.3% represented as t*T*_3_. This proportional distribution is nearly identical to that observed for wild (98 to 99%; Fair *et al.,* 2011) and captive (99.3%; West et al., 2014) bottlenose dolphins. This finding is also in line with the understanding in other mammalian species that *T*_4_ functions primarily as a prohormone reserve for intracellular conversion to the biologically active form *T*_3_ (for review, see Brent, 2012).

### Boto thyroid hormone concentration comparisons by age and sex

There were significant age differences in thyroid hormone levels, but these were not always consistent. For example, *T*_3_ and *T*_4_ were significantly increased in immature males compared with mature males but not between immature and adult females (non-pregnant, non-lactating females). For sex differences, significant differences in thyroid hormone concentrations were observed between immature male and female (*T*_3_, *T*_3_:*T*_4_) and adult male and female (*T*_4_, *T*_3_:*T*_4_) boto. These age and sex results differ from a study in wild bottlenose dolphins where significant differences in *T*_4_ between sex and age groups were largely due to juvenile males (Fair *et al.,* 2011). Similarly, immature females within both studies had the lowest *T*_3_:*T*_4_ ratio. In addition, our results are comparable with an earlier study in wild bottlenose dolphin whereby adult females had increased t*T*_4_ compared with adult males. However, unlike our findings, they also observed an increase in *T*_3_ in adult females compared with adult males. This observed difference may have been caused by the absence of controlling for lactating females in their comparisons with adult males, potentially increasing the overall mean *T*_3_ concentration in the female group (St. Aubin *et al.,* 1996). Research with belugas demonstrated a significant decrease with age in both t*T*_3_ and t*T*_4_ and significantly higher mean concentrations for male versus females; however, they did not directly compare sex differences between age classes. For the boto and beluga, the increase in *T*_3_ production observed in immature males versus immature females may reflect an increased or prolonged growth rate for dimorphically larger males whom require longer periods of time to reach sexual maturity (Robeck *et al.,* 2005; Martin and da Silva, 2006), and therefore, a prolonged growth hormone mediated increase in thyroid hormone production.

### Effect of reproductive state on thyroid hormone in adult female boto

We observed a significant 20% decrease in mean *T*_3_ concentration with no change in *T*_4_ during pregnancy as compared with non-pregnant non-lactating females. While our results were similar to a small number (*n* = 5) of captive beluga (Flower et al., 2015) and to a recent report in mares when controlling for season (Fazio *et al.,* 2016a), they contrast with reported results from captive bottlenose dolphins and many other mammalian species whereby thyroid hormone concentrations are significantly increased during pregnancy (Todini, 2007; West *et al.,* 2014). However, even within bottlenose dolphins, conflicting evidence exists, although results in wild animals were similar to ours in that t*T*_3_ and t*T*_4_ were reduced in pregnant females when compared to non-pregnant females (Fair *et al.,* 2011). For the captive bottlenose dolphin (West *et al.,* 2014), the significant increase detected in thyroid hormone during pregnancy compared with non-pregnant animals was primarily due to differences detected in the first trimester, whereby all thyroid hormones measured were increased. One possible explanation for the conflicting results observed between captive versus wild bottlenose dolphins and boto is that captive animals had multiple serial samples collected throughout pregnancy, while only single point samples were “randomly” collected from wild animals. Therefore, samples may have been clustered towards the end of the first trimester of gestation, a period when thyroid hormones are already decreasing towards the second trimester. Therefore, inaccuracy in trimester determination and/or clustering of samples in the late first trimester could theoretically result in missing peak concentrations of thyroid hormones observed during early pregnancy. For example, when weekly thyroid hormone concentrations were determined in horses, differences between pregnant versus non-pregnant controls were detected for t*T*_3_; however, these were primarily due to discrete significant increases during Weeks 7 and 12 post-ovulation (Fazio *et al.,* 2016b). If this pattern of secretion holds true for cetaceans, single point sampling from animals, which is typical for wild animal population studies, is more likely to results in misinterpretation of the thyroid hormone secretion pattern compared with repeated weekly measurements throughout gestation.

Clearly more work is required to understand normal cetacean thyroid function during pregnancy versus non-pregnancy, but what appears consistent between the boto and other cetacean and artiodactyl species (Todini, 2007; Todini *et al.,* 2007) is that thyroid hormone concentrations decrease from levels during early gestation to significantly lower concentrations during mid to late pregnancy. The mechanism for this change is believed to be due to a combination of factors that include increased metabolic need, an increase in production of thyroid binding globulin and direct pituitary stimulation from chorionic gonadotropins (Kennedy *et al.,* 2010). However, while these mechanisms have been advanced as explanations for increases observed during human pregnancy, they do not appear to apply directly to cetaceans. For example, the increase in thyroid binding protein is believed to be stimulated by early increase in estrogens, an event that does not occur in at least two odontocete species, bottlenose dolphins (Steinman *et al.,* 2016) and killer whales (Robeck *et al.,* 2017). In addition, no chorionic gonadotropin activity similar to that which is observed in humans (hCG) and horses (eCG) has been identified in cetaceans (Bergfelt *et al.,* 2012). Finally, the probable inappropriateness of comparing maternal thyroid hormone concentrations during pregnancy between species with different placentation should not be overlooked. For example, evidence suggests that little if any thyroxine crosses the placenta in mammals with epitheliochorial placentation (ruminants, cetaceans, etc.) after complete development of the maternal-placental barrier (mid to late gestation, Comline *et al.,* 1970; Hopkins and Thorburn, 1971), while significant transfer appears to occur for species with hemochorial placentation (primates). Therefore, species with active or passive transfer of thyroid hormones or their trophic precursors across the maternal-fetal barrier will have concentrations that reflect a combined homeostatic equilibrium of maternal and fetal requirements during the various stages and metabolic requirements of gestation, while in the former group, one would expect maternal concentrations to reflect metabolic demands for the dam, largely independent of fetal requirements. As the fetal thyroid develops, competition for circulating iodine, which readily diffuses across all types of placentas, may be the significant factor that results in decreased maternal circulating thyroid hormone hormones (Yarrington and Pearce, 2011) and maybe a partial explanation for the reduced thyroid hormone during late-term pregnancy in boto. Measurement of maternal iodine concentrations during pregnancy may provide evidence to support or refute this hypothesis.

The reduction in *T*_3_:*T*_4_ during late pregnancy provides some evidence to support the concept that iodine competition across the placenta, along with increased renal clearance of iodine may be, in part, the cause of this decreased ratio (Fisher, 1996). Because the *T*_3_:*T*_4_ ratio is believed to represent an indirect measurement of peripheral *T*_4_ deiodination to active *T*_3_ concentration (Todini *et al.,* 2007), a reduced ratio during pregnancy in the boto may reflect a decrease in maternal t*T*_4_ which is limited by iodine availability combined with an increased need for t*T*_3_. Accordingly, this phenomenon was not observed in captive or wild bottlenose dolphins (Fair *et al.,* 2011; West *et al.,* 2014), whereby their diet which consist of marine fish typically have an overabundance of iodine (Ridgway and Patton, 1971), whereas the fresh water fish food source on which the boto rely for are a magnitude lower in iodine content (Eckhoff and Maage, 1997; Fordyce, 2003). It has been noted that wild pregnant and captive bottlenose dolphins had increased thyroid gland mass compared with non-pregnant females (Cowan and Tajima, 2006; Kot *et al.,* 2012), and the goiterogenic effects of moderate iodine restriction during pregnancy in humans are well documented (Glinoer, 2004). In addition to possible iodine deficiency, the change in *T*_3_:*T*_4_ could simply indicate the inability of the thyroid to keep up with high metabolic demands of late pregnancy. Without further evidence, especially maternal iodine and TSH concentrations, and normal values for comparisons, the cause remains speculative.

Similar to lactating wild bottlenose dolphins (Fair *et al.,* 2011), we found a significant increase in t*T*_3_, and a non-significant decrease in t*T*_4_ (9%) compared with adult non-pregnant non-lactating females, and a corresponding significant increase in t*T*_3_:t*T*_4_ during lactation in the boto. These results are also compatible with an observed increase in thyroid size in wild lactating bottlenose dolphins (Cowan and Tajima, 2006) and an observed increase in thyroid volume during lactation in captive Indo-Pacific bottlenose dolphins (*Tursiops aduncus*, Kot *et al.,* 2012). Whether the observed thyroid hypertrophy was a residual effect from the known goitrogenic effects of pregnancy, as has been observed in humans (Glinoer, 2004), or a true response to increase demand during lactation for thyroid hormone is unknown.

Thyroid hormones appear to have both species-specific and time-specific alterations during lactation (Polat et al., 2014; Micke et al., 2015; Fazio et al., 2016a). Our results most resemble those of women during the post-partum period when a transient decrease in post-partum t*T*_4_ occurs in conjunction with an increased t*T*_3_ believed to be caused by the energetic demands of lactation (Weeke *et al*., 1982). The increase in *T*_3_:*T*_4_ also supports the concept that t*T*_4_ is being metabolized to t*T*_3_ at a faster rate than can be compensated for by thyrotrophic hormones or rate limiting effect of iodine availability. In support, a significant increase in the *T*_3_:*T*_4_ ratio paired with increased TSH concentrations is considered diagnostic for hypothyroidism in humans (Mortoglou and Candiloros, 2004). Without measures of TSH concentration in conjunction with thyroid hormone concentrations, it will be impossible to classify these significant changes as hypo- or hyper-thyroidism, but these changes illustrate and confirm the shifting demand on maternal thyroid function during these various reproductive states in the boto.

### Thyroid hormone concentrations in boto with successful versus non-successful pregnancies

Pregnant boto females, as diagnosed by ultrasonography, that were not observed with a calf the following year were assumed to have lost the fetus from an abortion, perinatal loss or had a calf that failed to thrive and died. We did not detect any differences in thyroid hormone concentrations during pregnancy between females that lost calves verse those with confirmed normal calves. These results contrast with those of captive bottlenose dolphins where a significant decrease in thyroid hormone concentrations during the second and thirrd trimesters in females that had perinatal loss was observed (West *et al.,* 2014). Our results may simply indicate that thyroid function was not a factor in the loss of the calves from these females or that the low numbers of females sampled would not allow enough sensitivity to detect significant changes. Given the potentially diverse array of etiologies for perinatal calf loss in wild populations, much larger sample sizes likely are needed to determine exact cause of such losses.

## Conclusions

Similar to many other wildlife species (Kersey and Dehnhard, 2014), little is known about thyroid function in any river dolphin species including the boto, and this study represents the first significant report. As the enviable loss or change in prey availability continues, and exposure to endocrine disrupting contaminants increases, both factors which have been implicated as affecting thyroid function (Fair et al., 2011; Hall et al., 2018), the data presented herein will provide a critical base of information from which the effects of these changes on the boto can be monitored. In addition, this information provides for comparisons with other species of river dolphins that are currently experiencing many of these anthropogenic threats to such a degree they are currently threatened with extinction (He et al., 2017).
